# Ginsenoside Rg5 Improves Radiation‐Induced Heart Injury via PPARG/PDK1/AKT1 Pathway

**DOI:** 10.1111/jcmm.70852

**Published:** 2025-10-15

**Authors:** Dao‐ming Zhang, Jun‐jian Deng, Hao‐yue Li, Yang Shen, Yang Li, Bing Wu, Yan Hu, Ya Zou, Qin Huang, Xi‐ming Xu

**Affiliations:** ^1^ Department of Oncology Renmin Hospital of Wuhan University Wuhan People's Republic of China; ^2^ CCU Renmin Hospital of Wuhan University Wuhan People's Republic of China; ^3^ Key Laboratory for Molecular Enzymology and Engineering of the Ministry of Education, School of Life Sciences Jilin University Changchun People's Republic of China; ^4^ Department of Obstetrics and Gynecology Renmin Hosptial of Wuhan University Wuhan People's Republic of China

**Keywords:** apoptosis, ginsenoside Rg5, PPARG/PDK1/AKT1, radiation‐induced heart disease

## Abstract

Ginsenoside Rg5 (G‐Rg5), a rare extract of ginseng, has proven to be valuable for a wide range of clinical applications. However, the role of G‐Rg5 in radiation‐induced heart injury is currently unclear. This study aims to explore the intervention effect and possible treatment mechanism of G‐Rg5 on radiation‐induced heart injury. We investigated the impact of G‐Rg5 on radiation‐induced heart injury through in vivo and in vitro studies. Network pharmacology was employed to explore potential key targets and molecular mechanisms. Various experimental methods were utilised to validate the effects of G‐Rg5 on the PPARG/PDK1/AKT1 pathway. G‐Rg5 alleviated radiation‐induced cardiomyocyte apoptosis and cardiac functional impairment. The expression of peroxisome proliferator activated receptor gamma (PPARG) was upregulated following G‐Rg5 treatment, thereby suppressing the transcription of phosphoinositide‐dependent protein kinase 1 (PDK1), a predicted target gene regulated by PPARG, to enhance AKT serine/threonine kinase 1 (AKT1) phosphorylation levels. The protective effect of G‐Rg5 against radiation‐induced heart injury was found to be compromised upon inhibition or knockdown of PPARG. The interaction between PPARG and PDK1 was confirmed by the results of chromatin immunoprecipitation and luciferase reporter assays. G‐Rg5, through the upregulation of PPARG expression, induces the transcription of PDK1 and subsequently enhances the phosphorylation levels of AKT1. Ultimately, this process plays a crucial role in mitigating cellular apoptosis and functional decline in radiation‐induced heart injury. Therefore, G‐Rg5 holds great potential as a therapeutic agent for radiation‐induced heart injury.

**Abbreviations**
AKT1: AKT serine/threonine kinase 1
Bax: Bcl‐2‐associated X protein
BCA: bicinchoninic acid Assay
Bcl‐2: B‐cell lymphoma‐2
CCK‐8: cell counting kit 8
CETSA: cellular thermal shift assay
ChIP: chromatin immunoprecipitation
CO: cardiac output
DMEM: dulbecco's modified eagle medium
DMSO: dimethyl sulfoxide
EF: ejection fraction
ELISA: enzyme‐linked immunosorbent assay
FS: fractional shortening
GAPDH: glyceraldehyde‐3‐phosphate dehydrogenase
GO: gene ontology
G‐Rg5: ginsenoside Rg5
IHC: immunohistochemistry
KEGG: kyoto encyclopedia of genes and genomes
LVEDP: left ventricular end‐diastolic pressure
mTORC1: protein kinase complex mechanistic target of rapamycin complex 1
PDK1: phosphoinositide‐dependent protein kinase 1
PPARG: peroxisome proliferator activated receptor gamma
PPI: protein–protein interaction network
qRT‐PCR: quantitative real‐time polymerase chain reaction
RIHD: radiation‐induced heart disease
SD: standard deviation
TUNEL: terminal deoxynucleotidyl transferase‐mediated dUTP nick end labeling
WHI: whole‐heart irradiation

## 1 Introduction

Radiation therapy, as a crucial clinical method for treating malignant tumours, has significantly improved the survival rates of cancer patients. However, it also inevitably leads to certain complications [1]. The heart is inevitably exposed to radiation during thoracic radiotherapy due to anatomical factors and the location of radiation. Radiation‐induced heart disease (RIHD) is a highly severe treatment‐related complication, characterised by pathological features such as oxidative stress‐induced damage and myocardial cell loss [2]. The clinical manifestations of RIHD encompass pericarditis, valve damage, myocardial infarction, arrhythmias and other related complications. RIHD ranks second in terms of both incidence and mortality among radiation therapy‐related complications. This highlights the paramount importance of prevention, diagnosis and intervention strategies for managing this condition.

Radiation—induced heart disease (RIHD) represents an intricate process underpinned by multiple interrelated mechanisms. In the initial stages, augmented oxidative stress instigates a cascade of biological processes. This includes damage to endothelial cells, the onset of acute inflammation and the induction of diverse forms of cell death such as apoptosis, autophagy and cellular senescence [3]. During the early chronic phase, surviving cells with sub—lethal injuries initiate several compensatory mechanisms. These involve the proliferation of endothelial cells and cardiac hypertrophy. However, once these compensatory mechanisms become exhausted, chronic inflammatory processes, fibrosis and endothelial cell senescence assume central roles in driving disease progression. Activated fibroblasts, in this context, over—produce collagen. The excessive deposition of collagen leads to thickening of the heart tissue, which in turn reduces its compliance and severely impairs cardiac function [4].

Ginseng, as a valuable medicinal herb mainly grown in East Asia, contains ginsenosides as its primary pharmacologically active components [5]. Among these ginsenosides, G‐Rg5 is one of the major compounds extracted from red ginseng and it has been confirmed that G‐Rg5 possesses an anti‐tumour effect that inhibits the proliferation of malignant tumour cells that induce DNA damage [6]. Modern pharmacological research has shown that G‐Rg5 can protect the heart by regulating arrhythmia, inhibiting myocardial cell hypertrophy and suppressing myocardial cell apoptosis [7, 8]. The inhibitory effect of G‐Rg5 on tumour cell growth is not only evident, but it also exhibits a cardioprotective potential, thereby positioning it as a promising pharmaceutical agent for the prevention and treatment of RIHD.

Despite the growing recognition of RIHD as a critical complication, current therapeutic options remain limited. Existing interventions primarily focus on symptomatic management, but lack targeted strategies to address the core mechanisms of radiation‐induced myocardial damage [9]. G‐Rg5 has shown promise in preclinical studies for cardioprotection, yet its specific role in RIHD and underlying molecular pathways remains unexplored. In our study, we identified the targets of G‐Rg5 and RIHD using various databases and predicted key targets and pathways for G‐Rg5's treatment of RIHD through protein–protein interaction network analysis and pathway enrichment analysis. We then validated these predictions using molecular docking and pharmacological experiments. This study aims to provide a rational theoretical basis for expanding the therapeutic applications of G‐Rg5.

## 2 Materials and Methods

### 2.1 Reagents

The dulbecco's modified eagle medium (DMEM) medium, Gentamicin and Trypsin used in this study were provided by Hyclone (Logan, Utah, USA). Fetal bovine serum was sourced from Gibco (Grand Island, USA). Dimethyl sulfoxide (DMSO) and Goat anti‐rabbit IgG/HRP were purchased from Sigma (Sigma‐Aldrich, St. Louis, MO, USA). The terminal deoxynucleotidyl transferase‐mediated dUTP nick end labeling (TUNEL) assay kit used was supplied by Roche (Roche Applied Science, Upper Bavaria, Germany) and the BCA protein quantification assay kit used was purchased from Sigma (Sigma‐Aldrich, St. Louis, MO, USA). The CCK‐8 assay kit, Cell apoptosis assay kit, Primary antibody dilution solution, Secondary antibody dilution solution, Protein Marker, RIPA, PMSF and cocktail were procured from Biyuntian (Shanghai China). The Protein loading buffer used in this study was supplied by Santa Cruz Biotechnology (Dallas, TX, USA), while the Rabbit anti‐mouse Bax antibody, Bcl‐2 antibody, Caspase‐3 antibody, beta‐catenin antibody, p‐AKT1 antibody, PPARG antibody and PDK1 antibody were sourced from Abcam (Cambridge, MA, USA). The reverse transcription kit used was supplied by ThermoFisher Scientific (CA, USA). Primer synthesis and siRNA design and synthesis were provided by GenePharma Corp. (Suzhou China).

**FIGURE 1:**
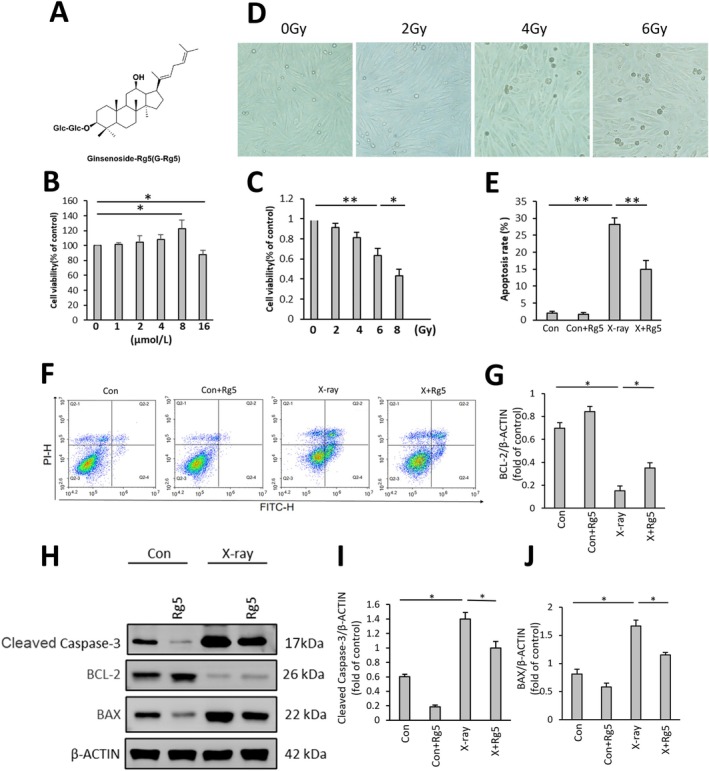
Ginsenoside Rg5 inhibited radiation‐induced apoptosis of H9c2. (A) Chemical structure of G‐Rg5. (B) Cell viability after 48 h of incubation with different concentrations of G‐Rg5‐containing culture medium. (C) Cell viability after 48 h of radiation in different doses. (D) Representative pictures of cells after different doses of radiation. (E) Bar charts of cell apoptosis rate. (F) Flow cytometry analysis of apoptosis rates in the Con group, Con‐Rg5 group, X‐ray group and X‐Rg5 group. (G–J) Western blot analysis of Cleaved Caspase‐3, BCL‐2 and BAX protein expressions. Data were normalised to the protein expression levels of β‐actin. Data are presented as the mean ± SD per experimental group (*n* = 6). *p < 0.05, **p < 0.01.

Ginsenoside Rg5 with a molecular formula of C42H70O12 and purity verified at 99.3% as shown in Figure 1A was purchased from Yuanye Bio‐Technology (Shanghai, China). GW9662 and Enalapril were also obtained from Yuanye Bio‐Technology (Shanghai China).

### 2.2 Cell Cultures

The H9c2 cell lines were cultured in the Central Laboratory of Wuhan University People's Hospital. Cells were incubated at 37°C in a 5% CO_2_ cell culture incubator until they reached approximately 90% confluence, following which subsequent procedures and related experiments were conducted. The cells were then cultured with Ginsenoside Rg5‐enriched medium at concentration gradients of 0, 1, 2, 4, 8 and 16 μmol/L for a duration of 48 h in an incubator. siRNAs obtained from GenePharma Corp. (Suzhou, China) were transfected into the cells using Lipofectamine2000 (5 μL/well) diluted to a concentration of 20 nM according to the manufacturer's instructions. The sequences utilised for PPARG siRNA were as follows: 5′‐UCCACACUAUGAAGACAUUTT‐3′ and 5′‐AAUGUCUUCAUAGUGUGGATT‐3′ (antisense). Non‐targeting siRNAs served as negative controls (NC). To inhibit PPARG expression, H9c2 cells were treated with GW9662 PPARG Inhibitor at a concentration of 20 μM when they reached approximately 50% confluence.

### 2.3 Animal Experiments

The C57BL/6 mice aged 6–8 weeks were procured from the Chinese Academy of Medical Sciences, Peking Union Medical College for conducting animal experiments in this study. These mice were housed at the Wuhan University People's Hospital Experimental Animal Center, where they were subjected to a 12‐h light–dark cycle and maintained under controlled conditions of temperature (22°C ± 2°C) and humidity (55% ± 5%). The ethical approval for performing animal experiments was obtained from the ethics committee of Wuhan University People's Hospital, ensuring compliance with the ‘Regulations for the Administration of Laboratory Animals’. All animal protocols conducted in this study received approval from the Animal Ethics Committee of Wuhan University People's Hospital (Licence No. 202300384; Approval time: May 10, 2023).

### 2.4 Establishment of Radiation‐Affected Cardiomyocyte Model

Well‐cultured H9c2 cells in the logarithmic growth phase were selected for this study. X‐ray irradiation was administered to the cells using a medical linear accelerator (Varian Trilogy, FL, USA) operating at an energy level of 6 MV/min, achieving a cell fusion rate of 80% or higher. Compensating membranes with a thickness of 1 cm were placed over the cells and the Source Skin Distance (SSD) was set to 100 cm. The cells were subjected to single irradiation doses of 2 Gy, 4 Gy, 6 Gy and 8 Gy separately. Subsequently, the irradiated cells were promptly returned to a temperature‐controlled incubator and cultured with G‐Rg5 (8 μmol/L) or DMSO (0.1%) for an additional 48 h for subsequent experiments.

### 2.5 Establishment of RIHD Mouse Model

After 1 week of adaptive feeding, the mice were randomly divided into six groups (*n* = 10/group): Sham group, Sham+Rg5 group (G‐Rg5, 25 mg/kg/day), WHI group, WHI + Rg5 group (G‐Rg5, 12.5 and 25 mg/kg/day) and WHI + Enalapril group (Enalapril, 2 mg/kg/day), which was designated as the positive‐control group due to enalapril's well‐established efficacy in heart injury. The RIHD model was established by performing whole‐heart irradiation (WHI). Mice were anaesthetised with an intraperitoneal injection of 1% sodium pentobarbital at a dose of 75 mg/kg. Once the mice were anaesthetised and securely immobilised, X‐ray irradiation was administered to the anterior heart regions using an X‐ray ionising radiation apparatus (Aolong Technology Co. Ltd., Dandong, China) at a single dose of 20 Gy with adjustment of a specialised radiation filter. In the Rg5 group and Enalapril group, corresponding doses of G‐Rg5 and Enalapril were administered intragastrically every day for 4 weeks after WHI. The Sham group and WHI group received the same volume of normal saline. The mice were euthanised for experimental measurements.

### 2.6 Western Blot

The hearts of mice were dissected and finely minced, followed by homogenisation in lysis buffer until complete dissolution. The resulting supernatant containing the buffer was collected. Alternatively, cells that had been previously washed were treated with lysis buffer to ensure complete cell lysis and the resulting supernatant was then collected. Protein extraction was performed and the protein concentration was quantified using a BCA assay (Sigma‐Aldrich, St. Louis, MO, USA). Denatured proteins were separated by sodium dodecyl sulphate‐polyacrylamide gel electrophoresis (SDS‐PAGE) and subsequently transferred onto a polyvinylidene fluoride (PVDF) membrane. The PVDF membrane was blocked with 5% skim milk at room temperature before incubation with an enzyme‐linked anti‐mouse IgG antibody. An ECL detection solution (Beyotime Biotechnology, Shanghai, China) was evenly applied to the PVDF membrane for visualisation of protein bands using an Odyssey CLx gel imaging system. Quantitative analysis of the bands was performed using Image J.

### 2.7 ELISA Assay

The target proteins in cells, including Bax, Bcl‐2, Cleaved Caspase‐3, p‐AKT1 and PDK1, were identified using enzyme‐linked immunosorbent assay (ELISA) kits (Biyuntian, Shanghai, China) according to the manufacturer's instructions.

### 2.8 Real‐Time Quantitative PCR

TRIzol reagent was used to extract total RNAs from cells or tissues and SuperScript RT kit was used to synthesise cDNAs. Subsequently, SYBR Premix Ex Taq II on a fluorescence quantitative PCR instrument was used to perform RT‐qPCR analysis. Primers for real‐time PCR were as follows: PPARG: 5′‐TCGCTGATGCACTGCCTATG‐3′ (Forward) and 5′‐GAGAGGTCCACAGAGCTGATT‐3′ (Reverse); glyceraldehyde‐3‐phosphate dehydrogenase (GAPDH): 5′‐ATCAAGAAGGTGGTGAAGCA‐3′ (Forward) and 5′‐AGACAACCTGGTCCTCAGTGT‐3′ (Reverse). With GAPDH used as internal reference genes, a 2^‐ΔΔCt^ method was employed to quantify the RNA expression levels.

### 2.9 Cell Viability Assay

The cells in logarithmic growth phase were harvested and seeded at a density of 5 × 10^4^ cells per well in a 96‐well plate. Following 48 h of incubation, each well was supplemented with 0.01 mL of CCK‐8 solution, followed by an additional 2‐h dark incubation period. Subsequently, the absorbance OD values of the cells were measured using an ELISA reader at a wavelength of 450 nm. In this study, ODa represented the OD values of cells in each experimental well, ODc denoted the OD values of cells in control group wells and ODb indicated the OD values of cells in wells containing only culture medium and CCK‐8. Myocardial cell viability was calculated using the following formula: [(ODa – ODb)/(ODc – ODb)] × 100%.

### 2.10 Apoptosis Assay

After digestion with trypsin without EDTA, cells were resuspended in 500 μL of Binding buffer. Subsequently, a 5 μL volume of Annexin V‐APC was added to the buffer and thoroughly mixed. After that, another 5 μL volume of PI dye solution was added to the buffer. Then, less than 1 h later, a flow cytometry analysis was conducted.

### 2.11 Transesophageal Echocardiography Examination

Mild anaesthesia was induced in mice through intraperitoneal injections of pentobarbital sodium. Subsequently, depilatory cream was applied to successfully remove the neck and chest hair of anaesthetised mice. A small amount of ultrasound coupling agent was then administered to the precordial area of each mouse, enabling acquisition of a two‐dimensional long‐axis image under ultrasound mode for assessment of their cardiac condition in an optimal state. Following this, ultrasound images were obtained and saved using M‐mode, with measurements conducted over three or more cardiac cycles. Relevant cardiac parameters such as cardiac output (CO), ejection fraction (EF), fractional shortening (FS) and left ventricular end‐diastolic pressure (LVEDP) were measured and recorded using M‐mode imaging. This examination aims to evaluate the cardiac functions of mice.

### 2.12 TUNEL Staining

After fixation in a fixative solution for 36 h, the cardiac tissues were dehydrated using a tissue dehydrator. Subsequently, tissue embedding was performed using a paraffin embedding machine. Following preparation and trimming of paraffin blocks, tissue sections with a thickness of 4 μm were obtained using a microtome. These sections were then floated out through a water bath and mounted onto glass slides. To ensure bubble‐free, flat and secure attachment of the tissue sections, control sections were carefully placed on the slides. The slides were promptly transferred to an oven for further processing before conducting TUNEL staining on the prepared tissue sections.

### 2.13 Bioinformatic Analysis

With a combination of the PubChem Compound sub‐library with Pharmapper, potential targets of Ginsenoside Rg5 were predicted. Through the GeneCard database, relevant targets of RIHD were obtained. Through Cytoscape 3.9.1 software, a ‘Drug Compound‐Targets‐Disease’ network was constructed, with network properties analysed. Matched target sets were input into the STRING database to build a protein–protein interaction network (PPI). Then, Gene Ontology (GO) enrichment analysis was performed to describe gene functions and Kyoto Encyclopedia of Genes and Genomes (KEGG) pathway enrichment analysis was carried out to explore the significantly enriched signalling pathways. JASPAR website was used for binding site prediction. This study employed AutoDock Vina 1.1.2 software for molecular docking.

### 2.14 Chromatin Immunoprecipitation (ChIP)

Chromatin Immunoprecipitation (ChIP) was performed using the ChIP Enzymatic Chromatin IP kit (GENMED). Cells were lysed with SDS buffer and subsequently, DNA was fragmented into 100–500 bp fragments using sonication. Subsequently, specific antibodies or normal mouse IgG were employed for DNA pulldown. Washing steps involved high‐salt and low‐salt buffers, followed by elution and cross‐linking of the DNA. The enriched sequences were evaluated through qRT‐PCR.

### 2.15 Luciferase Reporter Assay

The promoter region of the PDK1 gene was identified through the Ensemble website and based on predicted sites from the JASPAR database, we selected the sequence of the first 700 bp of the promoter. Subsequently, this sequence was recombined with the pGL3‐basic vector to generate a plasmid (pGL3‐PDK1‐promoter). For transfection, cells were transfected with 100 ng of PCDNA 3.1(+)‐PPARG plasmid, 100 ng of pGL3‐PDK1‐promoter plasmid and 50 ng of pRL‐TK plasmid using a DNA transfection reagent. Following transfection, cells were incubated for 48 h in a cell culture incubator. Luciferase activity was quantified using the Firefly‐Renilla dual‐luciferase reporter assay according to Promega's E1910 kit.

### 2.16 Cellular Thermal Shift Assay (CETSA)

The H9c2 cells were lysed on ice using RIPA buffer for 4 h, followed by five freeze–thaw cycles. Subsequently, the cell lysates were centrifuged at 12,000*g* for 15 min at 4°C to collect the supernatant. The obtained supernatant was divided equally and treated with either 8 μM G‐Rg5 or DMSO at a temperature of 25°C for a duration of 1 h. Following this treatment, the samples were divided into six equal portions and subjected to heating at temperatures of 50°C, 54°C, 58°C, 62°C, 66°C and 70°C for a period of exactly 2 min each. After heating, the samples underwent centrifugation at 12,000*g* for 15 min at 4°C and the resulting supernatants were collected and boiled with loading buffer. Finally, the expression levels of target proteins were determined through western blot analysis.

### 2.17 Data and Statistical Analyses

The data analysis was conducted using SPSS version 23. Quantitative data obtained during the experiments were presented as mean ± standard deviation (mean ± SD). Each experimental group underwent a minimum of three parallel repetitions for measurement. Independent sample *t*‐tests were employed to assess differences between any two groups, while one‐way analyses of variance (ANOVA) were used for comparing multiple groups. Statistical significance was determined at *p* < 0.05. GraphPad Prism 8 software was utilised to generate graphs and figures. By employing the aforementioned methodology, statistical analysis and visualisation of the experimental outcomes can be achieved.

## 3 Results

### 3.1 Intervention of G‐Rg5 Inhibiting Radiation‐Induced Apoptosis of H9c2 Cells

Various concentrations of G‐Rg5 (0, 1, 2, 4, 8 and 16 μmol/L) were employed in the experiment to determine the minimal effective concentration of G‐Rg5 on H9C2 cell viability. Within the low‐dose range, cardiomyocytes cultured in medium containing G‐Rg5 exhibited an upward trend in their survival rates (Figure 1B). However, at concentrations exceeding 8 μmol/L, cell growth was impeded, leading to decreased cell viability (87.67% ± 5.66%). Consequently, a concentration of 8 μmol/L for G‐Rg5 was chosen for subsequent investigations. To assess the impact of X‐ray radiation on myocardial cell viability, we utilised the CCK8 assay to measure cell survival rates. X‐ray radiation significantly reduced cell viability (*p* < 0.05) in a dose‐dependent manner (Figure 1C), and morphological changes characteristic of apoptosis can be observed under the microscope (Figure 1D). Since cellular viability dropped below 50% at an exposure dose of 8 Gy, rendering it unsuitable for further experiments, therefore we selected a radiation dose of 6 Gy for subsequent studies.

Furthermore, radiation‐exposed cardiomyocytes cultured with G‐Rg5 demonstrated a significant reduction in apoptosis rate with statistical significance (Figure 1E,F). Additionally, Western blot protein analysis was conducted to examine apoptosis‐related proteins. The results revealed that compared with the Con group, the Con‐Rg5 group displayed decreased expressions of Bax and Cleaved Caspase‐3 and increased expressions of Bcl‐2. On the other hand, the X‐ray group exhibited significantly higher expressions of Bax and Cleaved Caspase‐3 than those observed in the Con group. In contrast, the X‐Rg5 group showed lower expressions of Bax and Cleaved Caspase‐3 than those seen in the X‐ray group and higher expression levels of Bcl‐2 than that found within the X‐ray group. These findings indicate that Ginsenoside Rg5 can alleviate radiation‐induced apoptosis of H9c2 (Figure 1G–J).

### 3.2 G‐Rg5 Improving Apoptosis and Cardiac Dysfunctions of RIHD Mice

**FIGURE 2:**
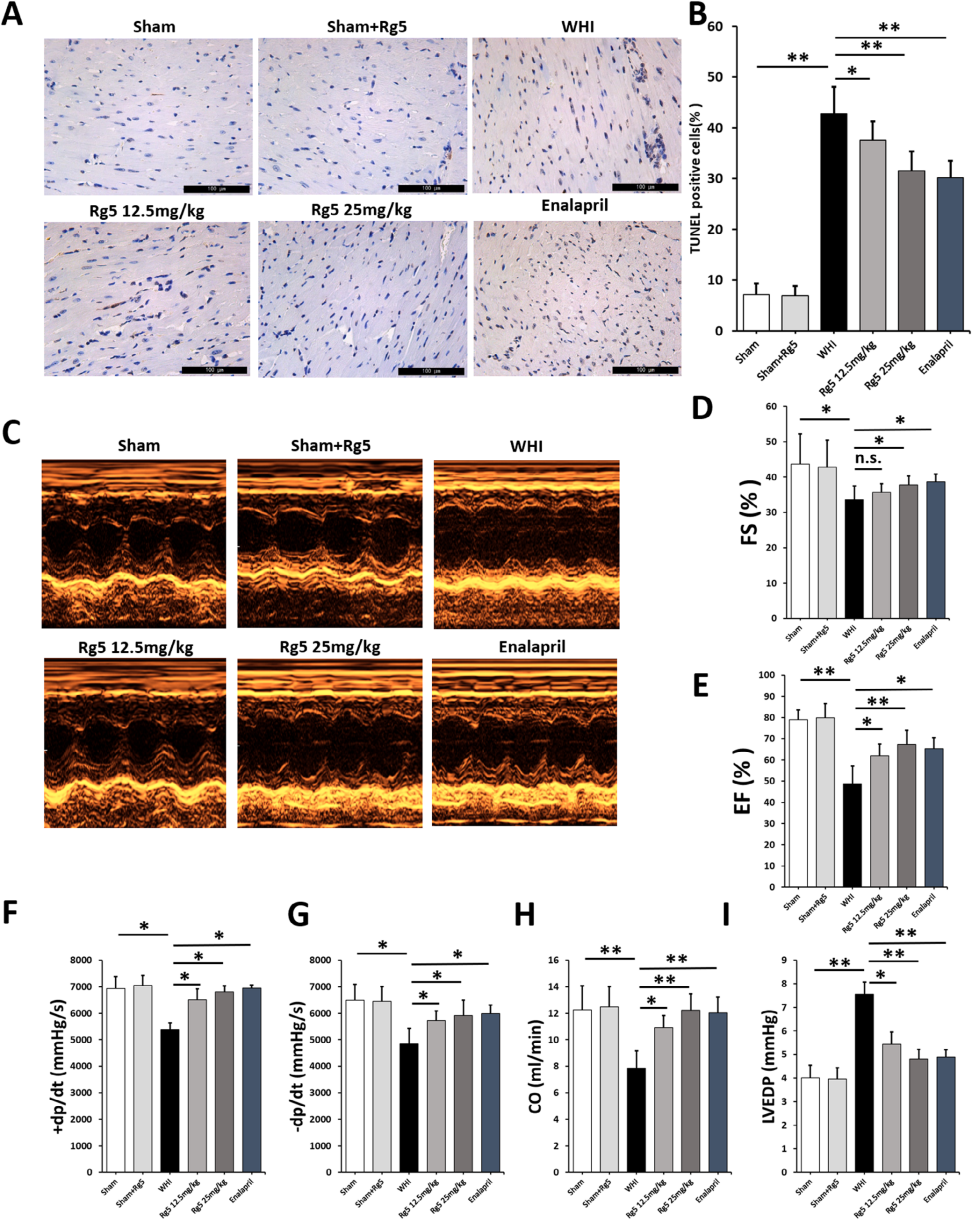
Ginsenoside Rg5 improved apoptosis and cardiac dysfunctions of RIHD mice. (A) Terminal deoxynucleotidyl transferase‐mediated dUTP nick end labeling (TUNEL) staining (×400 magnification) of mouse myocardial sections. (B) Quantification of TUNEL‐positive cells in heart tissue. (C) Representative M‐mode echocardiographic images of mice. (D) FS, (E) EF, (F, G) ±dP/dt, (H) CO and LVEDP in each group of mice. Data are presented as the mean ± SD per experimental group (*n* = 3 mice per group). *p < 0.05, **p < 0.01.

To validate the protective effect of G‐Rg5 on RIHD, we established a mouse model of RIHD with G‐Rg5 intervention in this study. Meanwhile, the mouse model of RIHD with Enalapril intervention served as the positive control group. Cardiomyocyte apoptosis was evaluated using the TUNEL staining method in the mouse model. The number of apoptotic‐positive cells significantly increased in mice treated with WHI compared to those in the Sham group. However, mice treated with G‐Rg5 in the WHI + Rg5 groups exhibited a noticeable dose‐dependent reduction in apoptotic‐positive cells (Figure 2A,B). These findings suggest that G‐Rg5 can alleviate radiation‐induced cardiomyocyte apoptosis in mice. Echocardiography was employed to assess cardiac functions (Figure 2C), revealing that RIHD mice treated with G‐Rg5 showed higher values for left ventricular FS (Figure 2D), EF (Figure 2E), maximum rate of left ventricular pressure rise or fall (±dP/dt) (Figure 2F,G) and CO (Figure 2H) compared to untreated RIHD mice without G‐Rg5 treatment. Furthermore, G‐Rg5 improved decreases in LVEDP induced by radiation exposure (Figure 2I). These results further confirm that G‐Rg5 can ameliorate cardiac dysfunctions observed in WHI mice.

### 3.3 Construction of Rg5‐RIHD Shared Gene Network ‘Drug Compound‐Targets‐Disease’

**FIGURE 3:**
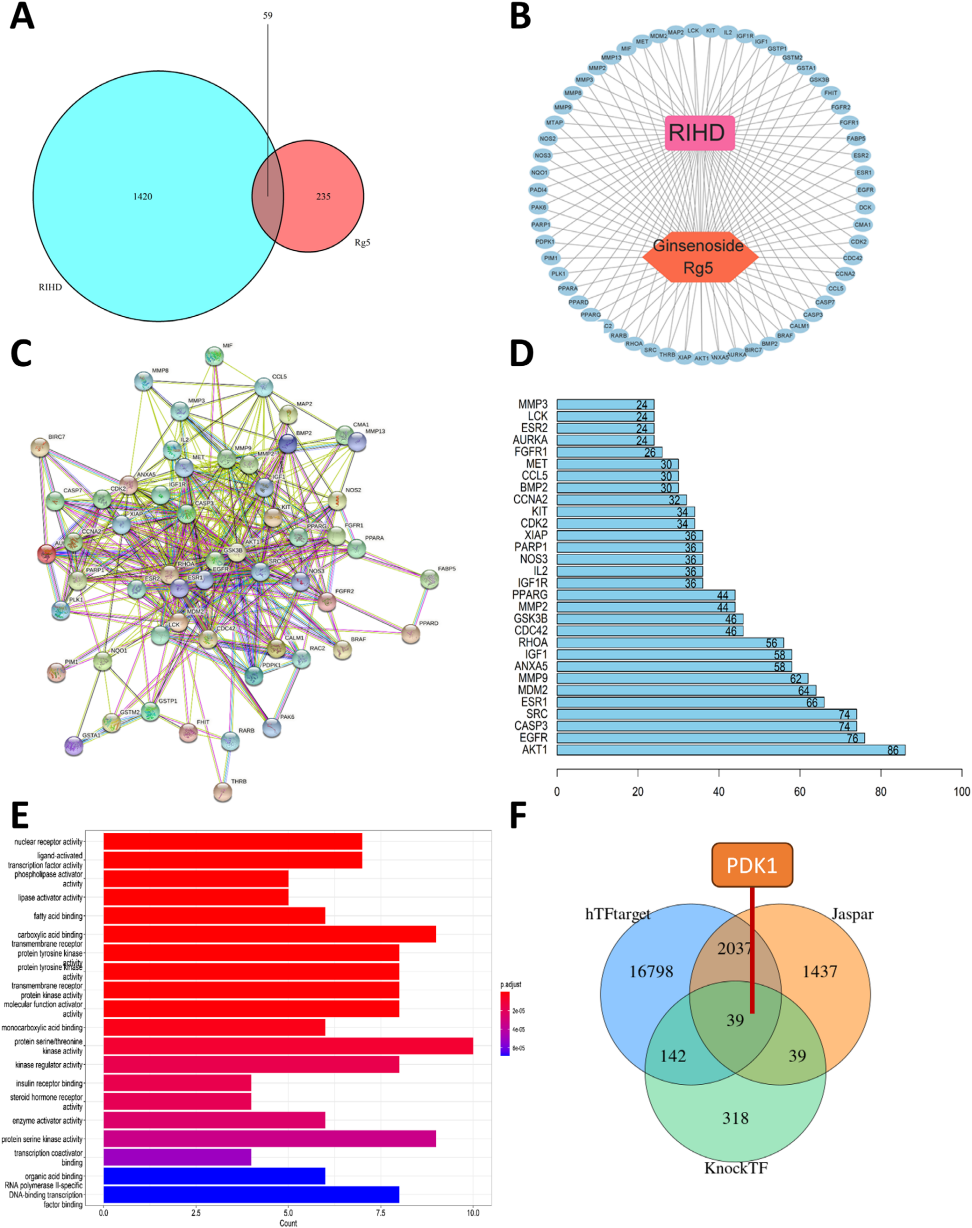
Network pharmacology analysis of Rg5‐RIHD. (A) Venn diagram of shared genes between Rg5 and RIHD. (B) Visual diagram of Ginsenoside Rg5 network ‘Drug Compound‐Targets‐Disease’. (C) Rg5‐RIHD protein interaction network. (D) Top 20 key targets predicted. (E) GO analysis of Rg5‐RIHD shared targets. (F) The Venn diagram prediction of PPARG target genes.

A total of 1479 target genes associated with RIHD were retrieved from the GeneCards database. Subsequently, these target genes were compared with the potential targets of G‐Rg5, resulting in the identification of 59 common targets (Figure 3A). Using Cytoscape 3.9.1 software, a visual representation depicting the network between G‐Rg5, its targets and RIHD was generated and displayed (Figure 3B). In this diagram, shared targets of G‐Rg5 and RIHD are represented by blue elliptical nodes, while RIHD and G‐Rg5 are depicted as pink rectangles and orange hexagons respectively. It clearly illustrates that G‐Rg5 employs a multi‐target approach for treating RIHD.

### 3.4 PPI for the Treatment of RIHD With G‐Rg5 and Its Functional Enrichment Analysis

By incorporating G‐Rg5 and RIHD's 59 shared target genes into the STRING database, we constructed a PPI for these genes (Figure 3C), without identifying individual proteins. Based on Count values, we selected the top 20 key target genes (Figure 3D). To investigate the functional annotations of these 59 shared target genes, we employed GO with a significance threshold of *p* < 0.05, resulting in a total of 98 enriched biological process terms. Among these terms, the most representative biological processes included nuclear receptor activity, ligand‐activated transcription factor activity, phospholipase activator activity, lipase activator activity, fatty acid binding, carboxylic acid binding, transmembrane receptor protein tyrosine kinase activity and others (Figure 3E). Our primary focus centred around PPARG among the major targets due to its involvement in various DNA transcription activities as a transcription factor. Using online prediction tools such as Tftarget, KnockTF and Jaspar to identify potential regulatory targets of PPARG, we discovered PDK1 which has been definitively confirmed to have a positive regulatory role in AKT1 phosphorylation (Figure 3F).

### 3.5 G‐Rg5 Binding Inducing PPARG Expression, Leading to an Increase in the Phosphorylation Level of AKT1

**FIGURE 4:**
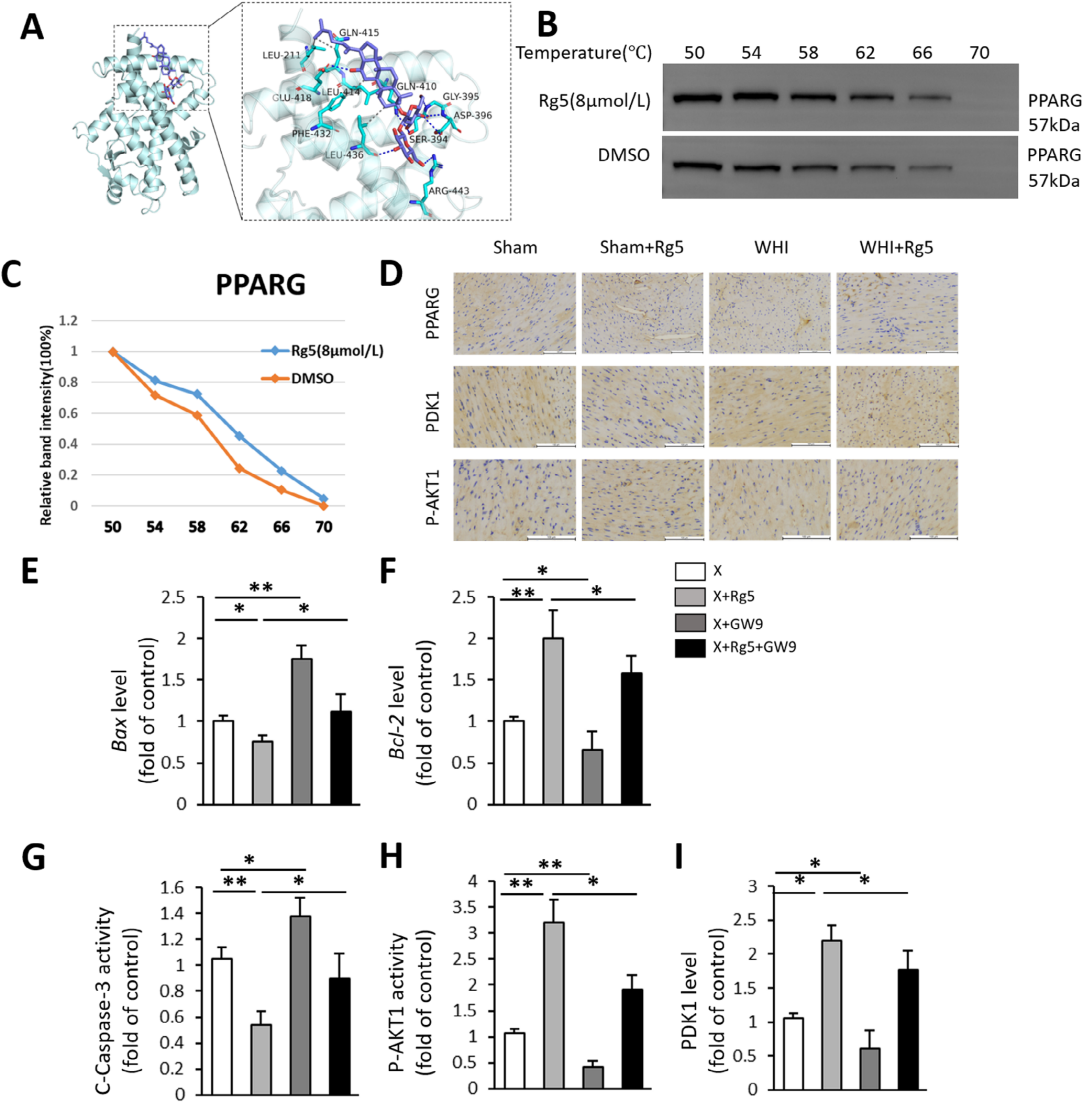
Binding of ginsenoside Rg5 induced PPARG expression, leading to increased phosphorylation of AKT1. (A) Results of molecular docking shows the interaction between G‐Rg5 and PPARG. Blue sticks represent small molecules, cyan cartoons represent proteins, yellow lines indicate hydrogen bonds and grey dashed lines represent hydrophobic interactions. (B, C) The protein expression of PPARG of CETSA. (D) Representative images of PPARG, PDK1 and p‐AKT1 immunofluorescence in heart tissue. Quantitative results of (E) Bax, (F) Bcl‐2, (G) C‐caspase‐3, (H) p‐AKT1 and (I) PDK1 activity through ELISA detection. Data are presented as the mean ± SD per experimental group. Three independent experiments were conducted for the quantification (E–I). *p < 0.05, **p < 0.01. Scale bar, 100 μm (D).

Molecular docking analysis was performed to investigate the interaction between G‐Rg5 and PPARG‐related proteins. The results unveiled a potential binding affinity between PPARG and G‐Rg5, with smaller negative affinity values indicating a higher probability of binding. Specifically, G‐Rg5 established hydrogen bonds with several residues on the PPARG protein, including ASP‐396, GLU‐418, LEU‐436, GLY‐395, GLN‐410, ARG‐443 and SER‐394. Additionally, hydrophobic interactions were observed between G‐Rg5 and LEU‐414, LEU‐211, LEU‐436, PHE‐432 and GLN‐415 residues on the PPARG protein. These interactions provided robust van der Waals forces to facilitate binding. The docking software assigned a relatively strong binding affinity score of −7.547 ± 0.11 kcal/mol for the interaction between G‐Rg5 and PPARG (Figure 4A). Furthermore, CETSA results demonstrated that as the temperature increased from 50°C to 60°C, G‐Rg5 enhanced the stability of experimental samples containing PPARG protein (Figure 4B,C). Overall, the findings suggest that in RIHD treatment, G‐Rg5 primarily targets the PPARG protein.

Subsequently, immunohistochemical analysis was performed on cardiac tissues obtained from the radioactive heart injury model to evaluate protein expression levels. The results demonstrated a decrease in PPARG expression and AKT1 phosphorylation within the heart tissues following radiation exposure. However, intragastric administration of G‐Rg5 to mice partially reversed these observed protein alterations (Figure 4D). To ascertain the crucial role of PPARG in mediating the protective mechanism of G‐Rg5 against radiation‐induced cardiac injury, we employed the PPARG Inhibitor GW9662 to suppress PPARG expression in myocardial cells. Our findings revealed that inhibition of PPARG expression weakened the protective effect of G‐Rg5 against apoptosis in radiation‐induced myocardial cells, as evidenced by ELISA detection of apoptosis‐related proteins (Figure 4E–G), along with concurrent suppression of AKT1 phosphorylation levels (Figure 4H).

### 3.6 The Upregulation of PPARG Leading to an Enhancement in the Transcription Levels of PDK1, Thereby Increasing the Phosphorylation Levels of AKT1 After G‐Rg5 Intervention

**FIGURE 5:**
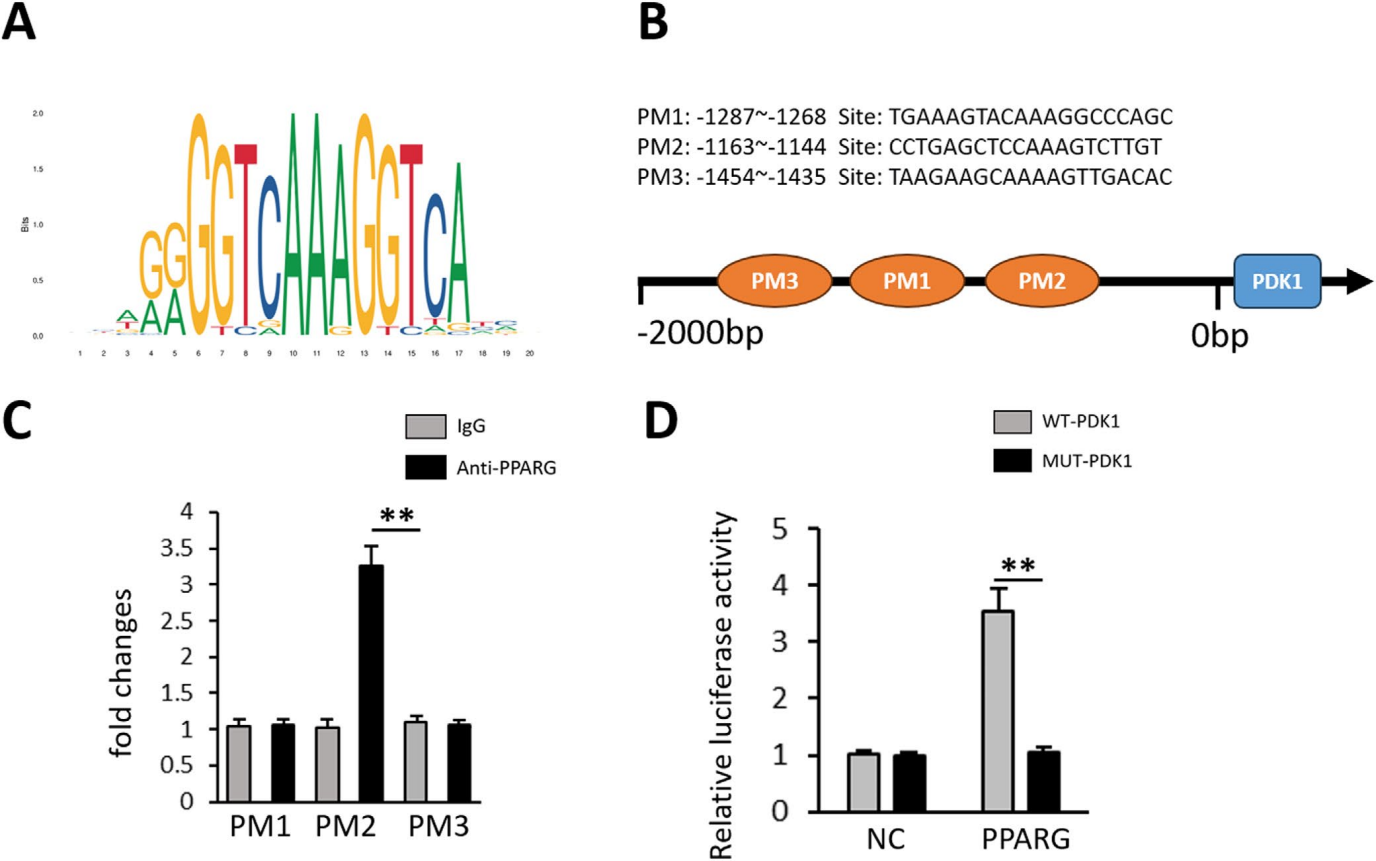
Upregulation of PPARG leaded to enhanced PDK1 transcript levels. (A) PPARG binding sites in PDK1 promoter predicted by JASPAR. (B) Schematic images of the potential PPARG binding sites in the promoter of PDK1. (C) ChIP analysis of PPARG occupancy at the PDK1 promoter. (D) Luciferase reporter assays. *p < 0.05, **p < 0.01.

To validate the predicted target gene PDK1 of PPARG through bioinformatics analysis, we performed immunohistochemistry (IHC) and ELISA analyses on heart specimens. The results demonstrated that G‐Rg5 intervention in a radiation‐induced cardiac injury model resulted in increased expression of PDK1 in cardiac tissues (Figure 4D). Furthermore, ELISA results indicated that the activation effect of G‐Rg5 on PDK1 in myocardial cells was partially reversed upon inhibition of PPARG expression (Figure 4I). To investigate whether PPARG functions as a transcriptional regulator for the PDK1 gene promoter, we utilised the JASPAR website to predict binding sites and identified three potential PPARG binding motifs named PM1–PM3 within the PDK1 promoter region (Figure 5A,B). Subsequently, ChIP‐qPCR was employed to experimentally validate these predicted binding sites in the promoter region. The ChIP‐qPCR results suggested that the PM2 site is likely to be the primary binding site for PPARG, while evidence supporting PM1 and PM3 sites is weaker (Figure 5C). In order to further confirm the interaction between PPARG and PDK1, we introduced mutations at the PM2 site and conducted a dual‐luciferase reporter gene assay. The outcomes revealed that while PPARG enhanced reporter gene activity for PDK1‐WT, it had no impact on PDK1‐MUT (Figure 5D), indicating an activating effect of PPARG on PDK1.

### 3.7 The Effects of G‐Rg5 on Radiation‐Induced Cardiomyocyte Apoptosis and the PPARG/PDK1/AKT1 Pathway Attenuated in PPARG Knockdown Cells

**FIGURE 6:**
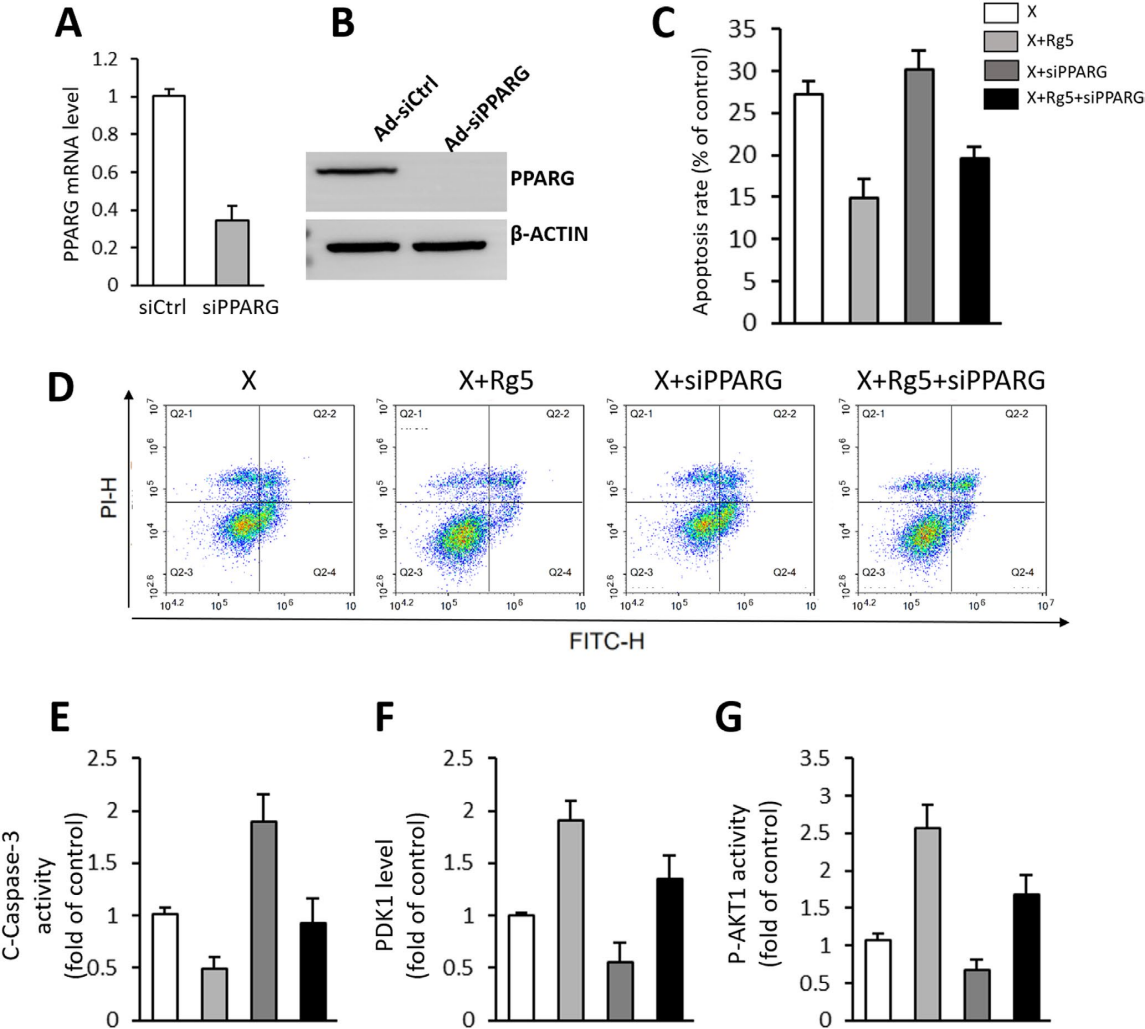
PPARG knockdown impaired the protective effect of ginsenoside Rg5 on radiation‐induced heart injury. (A) The expression of PPARG mRNA. (B) Western blot analysis of PPARG protein expressions. (C) Bar charts of cell apoptosis rate in the X group, X + Rg5 group, X + siPPARG group and X + Rg5 + siPPARG group. (D) Flow cytometry analysis of apoptosis rates. Quantitative results of (E) C‐caspase‐3, (F) PDK1, (G) p‐AKT1 activity through ELISA detection. Data are presented as the mean ± SD per experimental group (*n* = 6). *p < 0.05, **p < 0.01.

To further investigate the potential involvement of PPARG in mediating the cardioprotective effects of G‐Rg5, we utilised PPARG siRNA to silence PPARG expression in H9c2 cells. The results obtained from real‐time qPCR and Western blot analysis confirmed successful knockdown of PPARG, as evidenced by reduced levels of both PPARG mRNA and protein in cardiomyocytes (Figure 6A,B). Moreover, silencing PPARG attenuated the protective effect exerted by G‐Rg5 against radiation‐induced cardiomyocyte apoptosis (Figure 6C,D). Furthermore, ELISA analysis revealed that knockdown of PPARG hindered G‐Rg5's ability to decrease C‐caspase levels and increase both PDK1 and p‐AKT1 protein expression in the radiation‐induced injury cell model (Figure 6E–G).

## 4 Discussion

Existing therapeutic strategies for RIHD have made valuable contributions to alleviating clinical symptoms, though their scope in addressing the multifaceted mechanisms of radiation‐induced myocardial damage presents opportunities for further exploration. Our preclinical data suggest G‐Rg5 could offer a complementary approach for early intervention. This does not replace late‐stage therapies but addresses the unmet need for strategies to mitigate early pathological changes, potentially extending the clinical toolkit for RIHD management.

Ginseng, a widely utilised herbal medicine worldwide, has demonstrated significant efficacy in enhancing cognitive function, alleviating fatigue and weakness and improving attention deficits and physical performance [10, 11, 12, 13, 14]. G‐Rg5, an uncommon component extracted from ginseng through high‐temperature processing, exhibits diverse biological activities that make it a potential ingredient for treating various diseases. Currently, there is limited research on the role of G‐Rg5 in RIHD treatment; therefore, conducting relevant studies is necessary and meaningful. In our study, H9c2 cells cultured in a medium containing G‐Rg5 exhibited reduced apoptosis induced by radiation. Furthermore, intragastric administration of G‐Rg5 to mice with radiation‐induced cardiac injury resulted in notable improvement in heart function and significant reduction in myocardial apoptosis. This provides the first empirical evidence confirming the beneficial effects of G‐Rg5 in ameliorating radiation‐induced heart injury.

The feasibility and mechanism of G‐Rg5 in treating RIHD were explored through network pharmacology and bioinformatics analysis. Network pharmacology analysis identified a total of 298 potential target genes for G‐Rg5, indicating its multi‐target effect. Among them, fifty‐nine target genes associated with RIHD treatment were further identified. Analysis of the ‘disease‐drug shared target PPI’ network revealed that AKT1, EGFR, CASP3, SRC, ESR1, PPARG, among others, were significantly related to the effect of G‐Rg5. GO/KEGG analysis demonstrated that G‐Rg5 may intervene in the development process of RIHD through various biological activities and signalling pathways. These bioinformatics mining results provide clear guidance for our subsequent experimental efforts. We investigated changes in the phosphorylation levels of AKT1—a key player in a signalling pathway regulating diverse downstream effects such as cell survival, proliferation, growth and metabolism [15]. The findings indicated that radiation leads to decreased AKT1 phosphorylation in the heart; however, intervention with G‐Rg5 mitigates this reduction and enhances cardiac function.

AKT1 plays a pivotal role in cancer and its hyperactivation is closely associated with the pathogenesis and progression of various malignancies, including breast cancer, prostate cancer and lung cancer. Consequently, targeting AKT1 has emerged as a promising strategy for anticancer therapy [16]. Moreover, the activation of AKT1 exerts cardioprotective effects by attenuating myocardial damage caused by conditions such as myocardial infarction and heart failure [17]. Previous pharmacological studies on ginsenosides have demonstrated diverse regulatory effects on AKT expression depending on tissue type and disease context. For instance, G‐Rg5 was found to induce autophagy and caspase‐dependent apoptosis in NSCLC cells through inhibition of the PI3K/Akt/mTOR signalling pathway [18]. Additionally, G‐Rg5 induced apoptosis in human osteosarcoma cells via LC3‐mediated autophagy by suppressing the activation of PI3K/AKT/mTORC1 [19]. Notably, Kim et al. reported that G‐Rg5 stimulation led to Akt/mTOR phosphorylation while reducing Atrogin‐1 and MuRF1 expression levels, thereby promoting muscle regeneration and hypertrophy [20]. Furthermore, research findings suggested that ginsenoside Rg5 significantly inhibited hypoxia‐induced myocardial apoptosis through activation of the Akt signalling pathway [21]. The divergent effects of G‐Rg5 on AKT observed across different diseases have piqued our interest. We hypothesise that these discrepancies may arise from significant variations in transcriptional and translational regulation mechanisms governing AKT1 expression mediated by G‐Rg5 in distinct tissues ultimately leading to alterations in AKT1 phosphorylation levels.

Peroxisome Proliferator‐Activated Receptor Gamma (PPARG) was identified as a pivotal target gene, playing a crucial role in various biological processes. PPARG comprises an N‐terminal activation function domain (AF‐1), a DNA‐binding domain (DBD), a ligand‐binding domain (LBD) and a C‐terminal activation function domain (AF‐2) [22]. Collectively, these domains contribute to its diverse biological functions involved in the regulation of fatty acid synthesis, storage and breakdown, modulation of insulin sensitivity, as well as its implication in insulin resistance and diabetes development [23, 24, 25]. Research has also reported on the involvement of PPARG in cardiovascular diseases. A meta‐analysis conducted on eight independent expression datasets from the Gene Expression Omnibus (GEO) indicated that patients with myocardial infarction exhibited low expression levels of PPARG; furthermore, it was found to promote three myocardial infarction inhibitors (SOD1, CAV1 and POU5F1) while downregulating three markers (ALB, ACADM and ADIPOR2) for myocardial infarction protection [26]. Mechanistically speaking, the cardioprotective effects of PPARG include alleviating oxidative stress, inhibiting inflammatory responses, improving glucose and lipid metabolism, as well as antagonising apoptosis [27, 28, 29, 30]. In this study, we indeed observed inhibition of PPARG expression in the radiation‐induced cardiac injury model; however, intervention with G‐Rg5 increased the expression level of PPARG thereby mitigating radiation damage. Moreover, the protective effect exerted by G‐Rg5 was attenuated upon inhibition or knockdown of PPARG expression. Further molecular docking analysis along with CETSA revealed favourable binding affinity between G‐Rg5 and the PPARG protein. The binding interactions identified herein provide critical insights into G‐Rg5's mechanism of action. The stable interaction between G‐Rg5 and PPARG, mediated by hydrogen bonds and hydrophobic contacts, likely enhances PPARG's transcriptional activity.

To further elucidate the target genes regulated by PPARG as a transcription factor in its protective role against radiation‐induced cardiac injury, we employed online prediction tools to screen for potential PPARG target genes. The results revealed a favourable prediction score for the binding site between the promoter region of phosphoinositide‐dependent protein kinase (PDK1) gene and PPARG. It has been experimentally validated that PDK1 exerts an activating effect on AKT1 [31]. PDK1 activates AKT1 through two mechanisms: direct phosphorylation at Thr308 and recruitment of mTORC2, which phosphorylates AKT1 at Ser473, thereby promoting its activation [32, 33]. Notably, PDK1 forms heterodimers with both AKT1 and PKCα via interaction involving a PDK‐interacting fragment (PIF). This interaction occurs between the PIF pocket of PDK1 and the AGC binding partner's corresponding motif [34]. In this study, we observed a downregulation of PDK1 expression following PPARG inhibition. To further validate the direct role of PPARG in regulating PDK1 transcription, we conducted dual‐luciferase assays and ChIP experiments. The results demonstrated a significant alteration in luciferase intensity upon mutation of the predicted promoter binding site, indicating a noticeable change in the binding affinity between these two factors. The experimental data on PPARG‐PDK1 binding clarify the specific interaction mode and functional consequence of their association. This selective enrichment at PM2, compared to the weaker signals at PM1 and PM3, indicates that PPARG exhibits sequence‐specific recognition of the PDK1 promoter, with PM2 serving as the critical interaction locus. This result directly demonstrates that PPARG acts as a transcriptional activator of PDK1 and this activating effect is entirely dependent on the integrity of the PM2 binding site. The specificity of this regulation—restricted to the PM2 motif—highlights the precision of PPARG‐mediated transcriptional control in the context of PDK1 expression.

**FIGURE 7:**
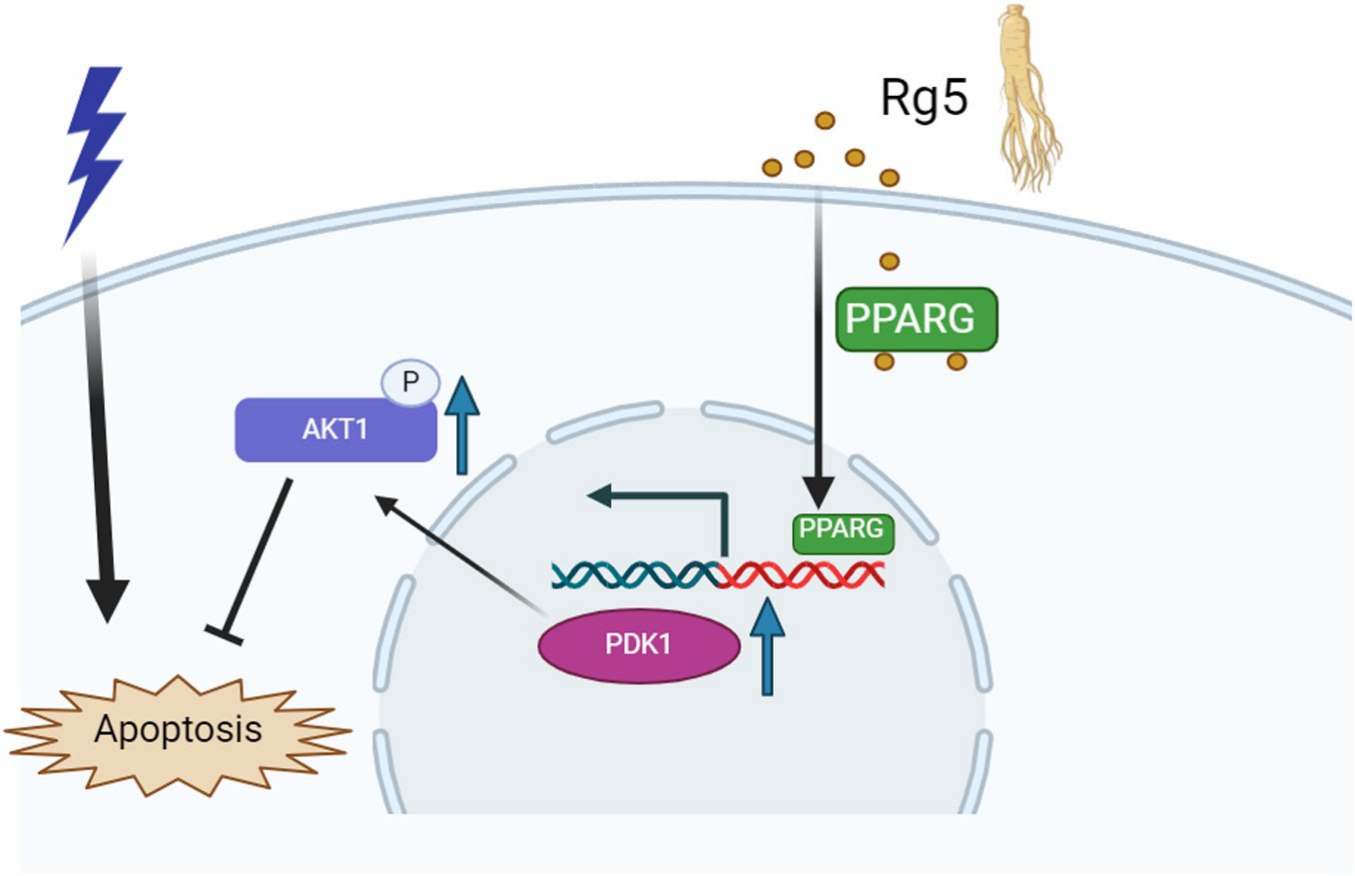
Ginsenoside Rg5 improves radiation‐induced heart injury via PPARG/PDK1/AKT1 Pathway.

Overall, our findings suggest that G‐Rg5 mitigates apoptosis and functional decline in radiation‐induced heart injury by upregulating PPARG expression, inducing PDK1 transcription and subsequently enhancing AKT1 phosphorylation levels (Figure 7). However, there are still limitations to be addressed in this study. The effects of G‐Rg5 on the RIHD mouse model extend beyond apoptosis; it may also impact fibrosis, autophagy and other forms of cell death. These mechanisms have not been investigated yet but hold substantial value for future research.

Future work should examine its cell‐type‐specific effects across cardiac cells and dynamic roles in acute, subacute and chronic RIHD phases. Optimising G‐Rg5's delivery, such as using cardiac‐targeted nanoparticles and testing combinations with existing therapies in large animal models could aid clinical translation. Exploring its efficacy in other cardiac pathologies and developing derivatives via structure–activity studies may expand applications. These efforts will enhance G‐Rg5's clinical value and deepen understanding of PPARG‐mediated cardioprotection.

## 5 Conclusion

We confirm that ginsenoside Rg5 induces transcriptional promotion of PDK1 by upregulating PPARG expression, subsequently leading to elevated phosphorylation levels of AKT1. Ultimately, this process plays a crucial role in mitigating cellular apoptosis and functional decline in radiation‐induced heart injury. These research findings provide valuable insights into the underlying mechanisms of ginsenoside Rg5 for treating radiation‐induced heart injury and offer directions for exploring its potential therapeutic effects.

## Author Contributions

**Dao‐ming Zhang:** conceptualization (equal), data curation (equal), methodology (equal), visualization (equal), writing – original draft (equal). **Jun‐jian Deng:** data curation (equal), formal analysis (equal), software (equal). **Hao‐yue Li:** data curation (equal), formal analysis (equal), investigation (equal), methodology (equal), validation (equal). **Yang Shen:** methodology (equal), software (equal). **Yang Li:** software (equal), visualization (equal). **Bing Wu:** methodology (equal). **Yan Hu:** methodology (equal). **Ya Zou:** methodology (equal). **Qin Huang:** data curation (equal), formal analysis (equal), resources (equal), software (equal). **Xi‐ming Xu:** conceptualization (equal), funding acquisition (equal), project administration (equal), writing – review and editing (equal).

## Conflicts of Interest

The authors declare no conflicts of interest.

## Data Availability

The datasets analyzed in this study and the original experimental results are available from the corresponding author upon reasonable request.
